# *Pediatric Critical Care:* Grand Challenges for a Glowing Future

**DOI:** 10.3389/fped.2014.00035

**Published:** 2014-04-30

**Authors:** Kanwaljeet J. S. Anand

**Affiliations:** ^1^Pain Neurobiology Laboratory, Department of Pediatrics, University of Tennessee Neuroscience Institute, University of Tennessee Health Science Center, Memphis, TN, USA

**Keywords:** child, infant, critical illness, trauma, pediatric intensive care unit

Pediatric critical care is a relatively new field, tracing its origins to the polio epidemics that killed large numbers of children, birthed by ongoing efforts in pediatric anesthesiology and neonatology, and nourished by parallel advances in pediatric pulmonology, cardiology, nephrology, adult critical care, general, cardiothoracic, neurosurgery, or other fields. Although not many pediatric intensive care units (PICUs) existed before 1980 (Table 1) (1–3), they now occupy a central position in the care of all hospitalized children and in their improved survival from all types of medical/surgical conditions. Despite an overwhelmingly clinical focus and limited avenues for disseminating research, the numbers of PICU-related publications have increased steadily over the past 20 years, currently hovering around 5000 reports per year.

**Table 1:** **Early history of pediatric ICUs**.

Year	Medical director	Institution
1955	Dr. Göran Hagland	Göteburg Children’s Hospital, Göteburg, Sweden
1961	Dr. Hans Feychting	St. Göran’s Hospital, Stockholm, Sweden
1963	Dr. J. B. Joly	Hopital St. Vincent de Paul, Paris, France
	Dr. I. H. McDonald	Royal Children’s Hospital, Melbourne, Australia
1964	Dr. G. Jackson Rees	Alder Hey Children’s Hospital, Liverpool, UK
1967	Dr. John J. Downes	Children’s Hospital of Philadelphia, Philadelphia, USA
1969	Dr. Stephan Kampschulte	Children’s Hospital of Pittsburgh, Pittsburgh, PA, USA
	Dr. James Gilman	Yale-New Haven Medical Center, New Haven, CT, USA
	Dr. Donald Clogg	Montreal Children’s Hospital, Montreal, Canada
1971	Dr. I. David Todres	Massachusetts General Hospital, Boston, MA, USA
	Dr. Alan Conn	Hospital for Sick Children, Toronto, Canada
1976	Dr. Peter Holbrook	Children’s National Medical Center, Washington DC, USA
	Dr. Mark Rogers	Johns Hopkins University Hospital, Baltimore, MD, USA
1980	Dr. Robert Crone	Children’s Hospital Medical Center, Boston, MA, USA
	Dr. Gregory Stidham	Le Bonheur Children’s Hospital, Memphis, TN, USA

*Pediatric Critical Care*, a section in the journal Frontiers in Pediatrics, seeks to disseminate the highest quality scholarly activity in this field, thus closing gaps between clinical practices and the high-level evidence supporting these practices. The four grand challenges include:

1. Fostering *innovation* in clinical medicine and technology.
2. *Translating* basic research into new diagnostic/therapeutic tools.
3. Defining short-term and long-term *outcomes*.
4. *Commitments* to research, training, and access to care.

## Fostering Innovation in Clinical Medicine and Technology

Recent discoveries elucidating the mechanisms of critical illness led to substantial advancements in pediatric critical care (1, 2), dramatically improving the outcomes of life-threatening illnesses or injuries in childhood. But now is not the time to rest on our laurels! Accelerating progress in multiple fields of biomedical and pharmaceutical sciences, imaging and computational sciences, and biomaterials and bioengineering sciences must be coupled with an unrelenting pursuit of basic science and clinical research to translate these discoveries into improving the care of very sick children. Pediatric intensivists must remain at the forefront of developing or evaluating novel technologies because of their unique perspectives gained from treating the whole patient and family; providing care at the end of life; and exposure to the entire ranges of demographics, medical/surgical conditions, and societal subgroups. Surveying the technological advances available for clinical application is impossible but two examples, regenerative medicine and personalized medicine, are mentioned here.

Innovation is rampant in the interdisciplinary fields of stem-cell therapy and regenerative medicine (4–8), which will significantly impact future treatments for organ failures, metabolic disorders, degenerative conditions, or the long-term sequelae of critical illness. Repair, replacement, or regeneration of various tissues or organs in critical illness is possible but complex (5–7), perhaps using combinations of several approaches including pluripotent stem cells, soluble molecules, genetic/tissue engineering, or advanced cell therapies (9–12). Using autologous bone marrow stem cells to restore cardiac myocytes (13–16) or neural stem cells for traumatic or hypoxic brain injury (17–19) will not only save lives but also considerably reduce the costs and side effects of managing chronic organ failure or neurodevelopmental sequelae.

Innovative advances in developmental biology and genetics/genomics will spawn “targeted” or personalized therapies for critical illness (20–22). The current explosion in genetic knowledge will help pediatric intensivists choose the best treatment among existing medicines based on a patient’s genetic, demographic, and environmental factors (21). Genetic adrenoceptor variants may dictate our choice of vasopressors and inotropes (23–25) or bronchodilators (26, 27), whereas opioid receptor variants may drive our choice of analgesics (28, 29). Pediatric intensivists will also have access to genetic tests revealing host susceptibility to specific infections, organ dysfunctions, or non-communicable diseases (30, 31). Studies on the human microbiome may help prevent sepsis in immune-compromised children (32) or tracheal infections in chronically ventilated patients (33).

Innovation will not come solely from these areas. Unique tools from clinical informatics can abstract patient data from electronic medical records and link these data with administrative, insurance, educational, or multi-institutional databases – providing the statistical power to address previously unanswerable questions (34, 35). Current researchers have the ability to cross-link genomic data with large clinical databases to generate genotype–phenotype correlations, thus revealing novel physiological pathways or therapeutic targets. Biopharmaceuticals designed by coupling “-omics” with combinatorial chemistry will allow them to identify previously unknown therapeutic targets. Implantable devices like drug-eluting stents, biodegradable polymers, or other devices will also play an important role in improving the clinical outcomes of PICU patients.

## Translating Basic Research into New Diagnostic/Therapeutic Tools

The route from laboratory research to newer therapeutics or diagnostics is long, difficult, and often fraught with regulatory mishaps or unforeseen obstacles (36–38). Examples, where obvious therapeutic targets with excellent pre-clinical data did not translate into safe/effective therapies (39–41), are well-known in critical care, such as immune-based therapies targeting the systemic inflammatory response syndrome or sepsis (42). These disappointments probably resulted from inapt extrapolations of mouse immunology to humans (43), reductionist biological principles (44), or inadequate consideration given to the multi-layered and intricately networked human immune system (45–47). Innovative leads to address the problems of *integration* may come from the Virtual Physiological Human project, which establishes a technological framework for studying the human body as a single complex system (48, 49). Large collaborative *in silico* models will help us to assemble and investigate the entire *human physiome*, with greater chances for drug discovery leading to therapeutic success than the experimental approaches used previously.

Translational research places greater emphasis on understanding the molecular underpinnings of pediatric critical illness and developing biomarkers with diagnostic/therapeutic relevance. Recent research shows biomarkers associated with specific organ dysfunctions in children, e.g., acute kidney injury (50, 51). Specific biomarkers for early organ injury can detect certain diseases at their earliest stages, before the onset of clinical signs or symptoms (51–53). Initiating supportive or therapeutic interventions when these diseases are easily treatable or preventable will improve clinical outcomes. Identifying sensitive and specific biomarkers will allow diagnosis, treatment, monitoring, and prevention guided by the patient’s molecular signals. Novel biomarkers built into clinical trials can serve as surrogate outcomes or predict the patient’s response to therapy.

Given the current environment of less research funding, greater regulatory hurdles, larger clinical studies, and high legal liability, most pharmaceutical companies are reluctant to develop new therapeutics, particularly for a niche market like children. This is a “Grand Challenge” in itself, but there is hope on the horizon. In an unprecedented move, 10 large drug companies and 7 non-profit organizations teamed up with National Institutes of Health (NIH) to develop drugs treating Alzheimer’s disease, diabetes, lupus, or rheumatoid arthritis (54). Such partnerships can be formed to tackle the widely prevalent life-threatening diseases like viral bronchiolitis or sepsis in children. To make this happen, however, pediatric intensivists must come up with visionary goals, eloquently articulate them to multiple stakeholders in society, industry, and government, and then deliver excellence in implementing the proposed approaches to tackle these conditions.

## Defining Short-Term and Long-Term Outcomes

Outcomes research relied on blunt instruments like mortality and morbidity, complications or secondary organ failures, or process outcomes like the length of ICU stay or hospital stay, direct or indirect costs, or quality-of-life parameters. Few studies have focused on measuring other relevant clinical outcomes to provide a more fine-grained assessment of patients’ response to therapy. Recently, however, newer sources of funding have stimulated greater interest in patient-centered outcomes, functional outcomes, technology/resource utilization, or behavioral and neurodevelopmental outcomes.

Pediatric intensivists must define the most suitable outcomes to test the primary or secondary hypotheses generated in their research, possibly based on *physiological* (e.g., heart rate variability, microcirculatory flow), *molecular* [e.g., glycoprotein KL-6 for ARDS (55), neutrophil gelatinase-associated lipocalin (NGAL) for kidney injury (51), shed CD163 for organ dysfunction (56)], or *imaging* biomarkers [e.g., apparent diffusion coefficient (ADC) using diffusion tensor imaging (57), oxygen extraction fraction (OEF) using positron emission tomography (58)]. Newer end-points can also come from clinical outcomes research, using newer methodologies based on comparative effectiveness, quality improvement, patient-centered outcomes, or population health research. The Patient-Centered Outcomes Research Institute has helped to define objective measures for patient-centered outcomes, such as continuity or satisfaction with care, decisional knowledge, conflict, or regret (59). These novel parameters must be included in classical study designs testing interventions in critically ill children.

Pediatric intensive care, like any other complex, high hazard enterprise (e.g., aviation) was identified as an environment where many adverse events occurred due to human errors (60–62). Investigations to reduce drug-related errors attributed to the “human factor” included, for example, computerized order-entry (63, 64), direct observation (65), or 24/7 availability of clinical pharmacists (66). However, isolated or piecemeal approaches may have a limited or short-lived effect in reducing human errors unless a safety-based culture is created in the entire hospital. Although initially resource expensive, this multifaceted approach leads to substantial reductions in serious adverse events, preventable harm, or hospital mortality, with some improvements in the safety culture (67). Researchers should explore the possibilities to improve future outcomes in the PICU using quality improvement science to prevent human errors (68).

Long-term functional or psychological outcomes following PICU admission were neglected in many previous studies in children. Examples from cancer and congenital heart surgery have showed the importance of evaluating long-term clinical outcomes (69–71), examining the functional status of children in their home, school, or hospital environment (72). Other long-term outcome measures may include neurodevelopmental or other assessments, but these methods are time consuming, apply to narrow age ranges, and may require specialized training. Newer measures must be objective, relevant, and measure what they are designed for with high sensitivity, specificity, and accuracy, while having strong psychometric properties. The use of disease-based patient registries (73), smart-phone technology, internet access, and social media will allow us to assess long-term clinical outcomes like never before (74, 75). The challenge is to develop innovative outcome measures using these tools to effectively assess the long-term consequences of critical illness or PICU therapies in children (76).

## Commitments to Research, Training, and Access to Care

Drug discovery or device development was previously funded by large investments from industry. This paradigm is changing, with increasing costs of drug development, augmented risks of failure in a difficult regulatory environment, unfamiliar drug targets, and increased legal liability, which have reduced the incentives to develop newer agents. Increasingly, biotech or other startup companies with low overhead costs and limited liability are developing drugs/devices, using R&D funds from federal granting agencies, philanthropy, crowdsourcing, or other resources (77, 78). To drive their research agenda, pediatric intensivists must actively collaborate with these companies and their low-cost efforts to test new products. The onus for validating new targets and translating basic science discoveries into commercially viable products is shifting increasingly from industry to academia (77). Academic faculty must forge mutually beneficial partnerships with the biotech industry, to advance their discoveries into new drugs for critically ill children. Early career investigators can develop innovative ways to collaborate with industry, by obtaining specialized molecules or reagents, bioengineered animals, or advanced training at these startup companies.

**Figure 1:**
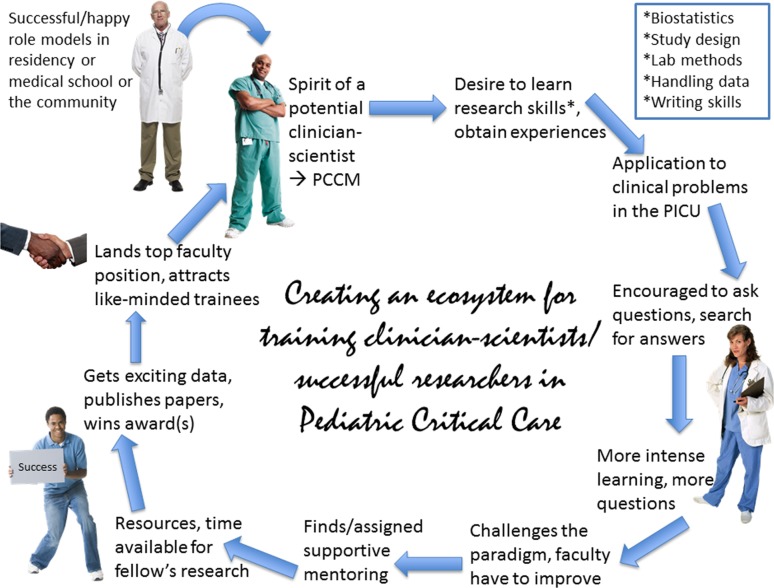
**Research-oriented trainees thrive in a challenging and learning environment**. Such an environment inspires, nurtures, and prepares trainees to devote their entire career in the pursuit of new knowledge in the basic sciences and/or its clinical applications, or other fields of enquiry. This flow-chart represents the life cycle of such a trainee in a research-oriented department. PCCM, Pediatric Critical Care Medicine; PICU, Pediatric Intensive Care Unit.

The pipeline of creative innovators in pediatric critical care will depend on the type of trainees we attract and the research training we offer; both factors are somewhat interdependent (79, 80). The challenge is to create an environment that fosters the curiosity, drive, and ambitions of trainees in pediatric critical care. Few departments have created an ecosystem that fosters consistent and sustained success in training new clinician scientists (Figure 1). Without wider commitment, dedicated time, and resources for research, the future growth of our specialty will be stunted and impoverished (79, 81). Programs such as the NIH-funded Pediatric Critical Care Scientist Development Program or training grants held by pediatric intensivists at other institutions provide important resources. Commitment to a clinician scientist’s career requires intense focus, strong mentorship, and opportunities for scientific growth even after training (82, 83). Trainees suited for health services or clinical research, educational research, or other scholarly activities can also provide valuable resources to the specialty.

A shortage of pediatric intensivists exists even in developed countries, with limited coverage in rural or remote areas. Regionalized PICU care increases coverage and controls costs, although for-profit hospitals often set up PICUs because they support a variety of other pediatric programs and services. Deficits in services and infrastructure for PICU care are more acute and widespread in resource-poor nations or international areas with armed conflict. Ensuring that all critically ill children get access to high-quality multidisciplinary intensive care is a huge challenge! Advances in telemedicine and transport medicine are now extending pediatric intensive care to some remote areas (84–86). Remote access to ICU monitors, real-time imaging, live video stream, and electronic medical records via high-speed internet connections allow pediatric intensivists to participate in the care of children located remotely (85, 86). Administrative hurdles in terms of medical licensing, patient privacy, malpractice liability, insurance coverage, and reimbursement procedures still need to be overcome in some healthcare markets (87). However, the clinical outcomes of remotely managed patients have not been reported, whereas recent studies show that patients requiring resuscitation or mechanical ventilation had improved outcomes when pediatric intensivists provided in-house coverage (88–90).

Research and policy changes to overcome these obstacles, together with alterations in clinical attitudes, approaches, and outcomes, will provide a rich milieu for research in pediatric critical care knowledge exchange and implementation science (91). Implementation science investigates the behaviors of healthcare professionals, administrators, patients, or other stakeholders as key variables in the uptake, adoption, and implementation of evidence-based interventions (67, 92). It can address major bottlenecks (social, behavioral, economic, and management) that impede the effective implementation of current evidence, test new approaches to improve healthcare, or determine causal relationships between an intervention and its clinical impact.

## Conclusion

The cumulative burdens of critical illness among children in developing and developed countries give us ample opportunities for research to prevent death, disability, and other limitations that keep children from reaching their full potential. Starting in this Year of the Horse, to be successful in preventing or managing critical illnesses that affect children today, researchers must harness and drive the four horses of *innovation, translation, outcomes*, and *commitments* sketched above. The pages of *Pediatric Critical Care* are eager to record their exploits and glory for posterity.

## Conflict of Interest Statement

The author declares that the research was conducted in the absence of any commercial or financial relationships that could be construed as a potential conflict of interest.
